# Placebo response varies between different types of sham acupuncture: A randomized double‐blind trial in neck pain patients

**DOI:** 10.1002/ejp.1924

**Published:** 2022-02-19

**Authors:** Dian Zeng, Xiaoxia Yan, Hongmei Deng, Jiemei Li, Jiaxin Xiao, Jiawei Yuan, Jianpeng Huang, Nenggui Xu, Wenbin Fu, Jianhua Liu

**Affiliations:** ^1^ Acupuncture Research Team The Second Affiliated Hospital of Guangzhou University of Chinese Medicine Guangzhou China; ^2^ South China Research Center for Acupuncture Guangzhou University of Chinese Medicine Guangzhou China

## Abstract

**Background:**

In prospective experimental studies of neck pain patients, it is difficult to determine whether responses to sham acupuncture differ from responses to real acupuncture due to the heterogeneous methodologies in control/sham interventions. Here we aim to compare the specific and nonspecific effects of electroacupuncture with four types of sham acupuncture.

**Methods:**

In this double‐blind, sham‐controlled study, we randomly assigned 175 patients with neck pain to receive 10 sessions of electroacupuncture, shallow puncture, nonacupoint deep puncture, nonacupoint shallow puncture, or nonpenetration acupuncture. We used the Northwick Park Neck Pain Questionnaire (NPQ) as our primary outcome, and Short‐form McGill Pain Questionnaire, visual analog scale (VAS), and Pain Threshold as secondary outcomes to measure the changes from baseline to a 3‐month follow up.

**Results:**

All groups, except nonacupoint shallow puncture, had significant improvement in all outcome measurements. Electroacupuncture only showed superior improvements than the shallow puncture, nonacupoint shallow puncture, and nonpenetration groups when compared using the NPQ and VAS scale (*p < 0.001). Interestingly, the nonacupoint shallow puncture produced even less placebo response than nonpenetration acupuncture.

**Conclusion:**

Our study demonstrates the high variability of placebo response among different types of sham controls depending on the depth of needle insertion and the puncture location. An important implication of our results is nonacupoint deep puncture produced similar analgesic effects as electroacupuncture. Our study may shed a new light on the predominant underlying mechanisms among different types of sham acupuncture controls, which can help with interpreting the effect of acupuncture in other studies.

**Trial registration:**

Chinese clinical trial registry (ChiCTR‐IOR‐15006886).

**Significance:**

This study compared the observed specific and nonspecific analgesia effect in four different types of sham acupuncture stimulation with neck pain patients, assessed by four outcomes. Although all of the sham controls produced significant reduction in neck pain, electroacupuncture had superior significant improvement. Importantly, placebo responses differed significantly between the sham controls and responses were inconsistent according to different outcome assessments. This study emphasizes the importance of taking into consideration which sham control and method of outcome measurement were used in a pain research study when evaluating its results.

## 1 INTRODUCTION

Despite 70% of people experiencing neck pain in their life time, many chronic or recurring, current treatment regimens for pain are ineffective for many individuals (Fejer et al., 2006; Guzman et al., 2009; Murray et al., 2015). As a traditional therapeutic approach, acupuncture has been found to induce the release of endogenous opioids and relieve pain by modulating brain regions and networks associated with pain perception (Cao et al., 2020; Lin et al., 2020). It has been shown that acupuncture treatment is particularly effective for upper body musculoskeletal pain, especially neck pain, in a previous meta‐analysis (Vickers et al., 2018).

However, acupuncture remains a controversial treatment for neck pain (Madsen et al., 2009; Seo et al., 2017). Some studies conclude that acupuncture is all placebo effect. Vase et al. (2016) emphasized that the placebo analgesia effect in study of pain is considerably large, which led to the difficulties of establishing treatment efficacy. For several years, placebo effects have been dismissed and considered a nuisance in trials despite the fact that clinicians and other health care practitioners have known that these effects can and do result in improvements of clinical symptoms. Unlike a placebo pill, sham acupuncture as a type of physical placebo, necessarily involves touching and administration which may cause other types of nonspecific physiological effects to occur in addition to placebo response. Hence, heterogeneous methodologies in control/sham interventions, short follow‐up times, and different outcome assessments in different clinical studies led to the inconclusiveness surrounding the efficacy of acupuncture as a treatment for neck pain.

The use of different types of sham acupuncture has been a critically contentious issue. Previous systematic reviews conclude that the limited benefits of real acupuncture were observed when it was compared with deep needling at nonacupoints, nonpenetrating needling, and superficial needling (Chen et al., 2019). Thus, the specific efficacy of acupuncture in neck pain varies from negligible to clinically significant. Vickers’ group pointed out that the effect sizes of acupuncture were smaller for sham‐controlled trials with penetrating needles (Vickers et al., 2018). Although the noninsertion controls produce the best results compared with penetrating sham controls, they may have a higher chance of blinding failure (Chen et al., 2016). It is critically important to evaluate which type of sham acupuncture is more appropriate for clinical trials.

Another factor which may influence the acupuncture effect is the method of outcome assessment. Despite visual analog scale (VAS) being the most commonly used for self‐report measurements (Dworkin et al., 2005), it has been questioned by Vickers that VAS may magnify the placebo effect (Vickers et al., 2018). Thus, patient‐reported outcome measures, like Northwick Park Neck Pain Questionnaire (NPQ), which evaluate the pain, emotion, and pain‐related disability together, have been proposed as a critical component for evaluating and monitoring pain (Bobos et al., 2018). In this study, we compared the electroacupuncture analgesia effect under different types of assessments. We used NPQ, VAS, Short‐form McGill Pain Questionnaire (SF‐MPG), and Pain Threshold for outcome assessments. Our primary aim is to analyze the placebo response under different types of sham intervention.

## 2 METHODS

In this randomized, double‐blind study, neck pain patients who were aged over 18 years‐old and diagnosed with neck pain for at least 3 months were recruited. Participants were excluded if they had: an average Visual Analog Scale (VAS) score of less than 3 in the past week; episodes of pain lasting less than 30 min; less than one episode of neck pain per week; received acupuncture treatment within 3 months; a history of cervical intervertebral disc herniation, taking corticosteroids, narcotics, muscle relaxants, or herbal medicines for pain control; skin infection; clotting disorders; previous acupuncture treatment for neck pain; other unstable conditions (e.g., heart failure, terminal stage of cancer) or pregnancy. All enrolled participants provided written informed consent. This study was conducted at Guangdong Provincial Hospital of Traditional Chinese Medicine, and was registered at the “Chinese clinical trial registry” (ChiCTR‐IOR‐15006886). We also published our study protocol at Trails (Yang et al., 2017).

All participants completed a 1‐week pain diary for pre‐screening. Only those with average pain levels of at least 3 were randomly assigned to the five groups via website (http://www.randomization.com) in random blocks of 3, 6, or 9. Participants, outcome assessors, and data analysis were blinded. The randomization was then concealed in sealed envelopes and was only opened by an acupuncturist who provided the treatment before the first session of stimulation. Each group (electroacupuncture group, shallow puncture group, nonacupoint deep puncture group, nonacupoint shallow puncture group, and nonpenetration group) was evaluated in four outcome assessments at baseline, after 5 sessions of treatment, after 10 sessions of treatment, and at the 3‐month follow‐up visit.

### 2.1 Intervention

**FIGURE 1:**
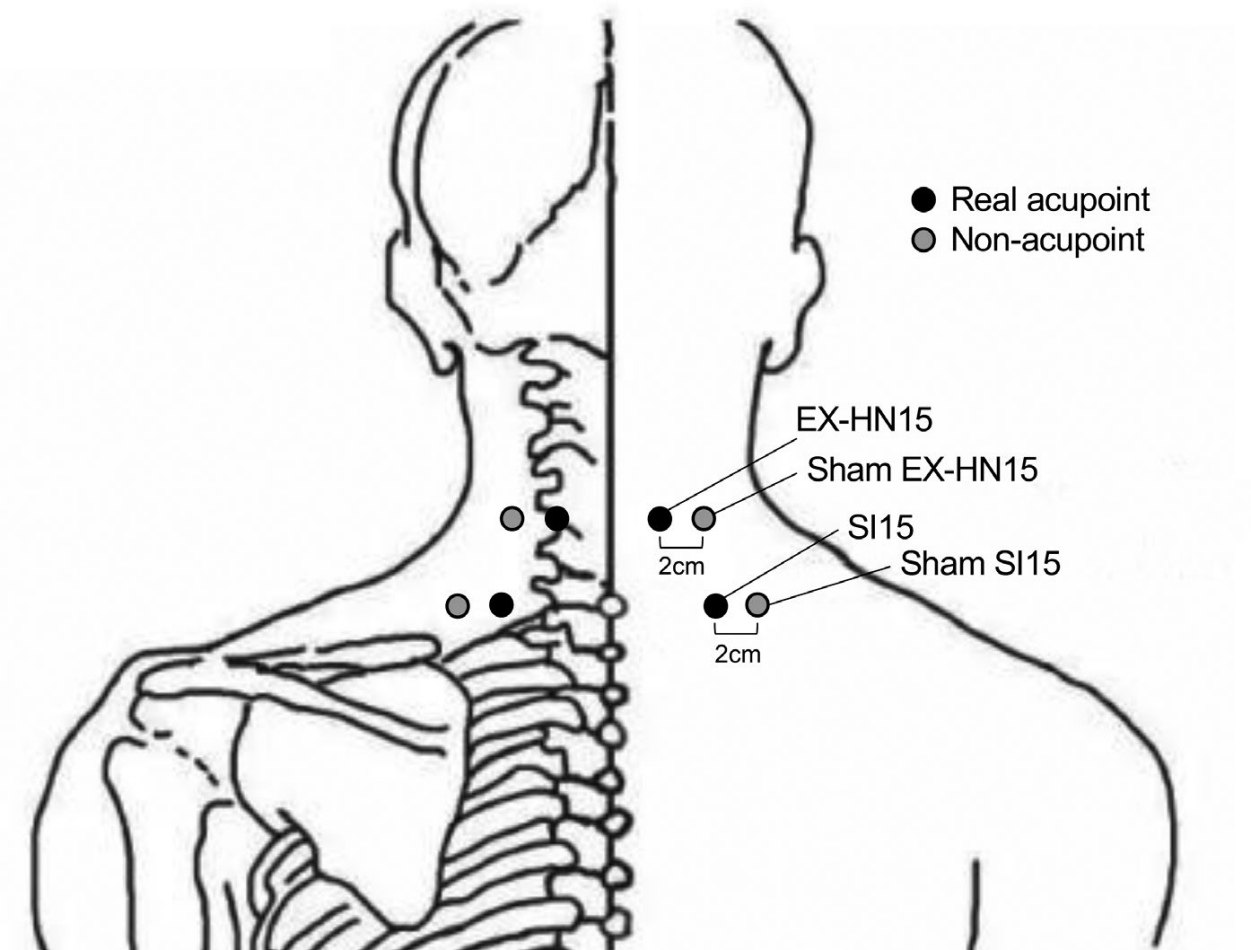
Location of acupoints

After baseline assessment, participants received 10 sessions of treatments in 3 weeks. Each session lasted about 30 min. The acupoints were bilateral Jingbailao (EX‐HN15), located on the neck, 2 cun superior and 1 cun lateral to *Dazhui*‐GV14 (1 cun ≈ 25 mm), and Jinzhongshu (SI15), located on the back, 2 cun lateral to the lower border of the spinous process of the seventh cervical vertebra (C7). Nonacupoints were located 2 cm lateral to the acupoints (EX‐HN15 and SI15) (Figure 1). The acupoints were selected based on our previous pilot study (Liang et al., 2011), which are the two major local acupoints for neck pain treatment. We only selected two acupoints in order to minimize the risk of unblinding.

For the electroacupuncture group, after the needle was inserted 0.5 cun (≈12.5 mm) into the skin at the acupoints, manipulations of twirling, lifting, and thrusting were performed on all needles to achieve the “de qi” sensation, which is composed of sensations of soreness, numbness, distention, heaviness. “De qi” is the essential factor for acupuncture effect (Zhou et al., 2011). The electroacupuncture stimulation had a continuous wave of 50 Hz and a current intensity of 1 to 5 mA, depending on each individual's tolerance, by a Han's Acupoint Nerve Stimulator (HANS‐200A). The needle for the nonacupoint deep puncture group was inserted as deeply as the needles used in the electroacupuncture group, but without any manipulation. For the two shallow puncture groups (both acupoint and nonacupoint), the needle was inserted less than 0.2cm into the skin. For the nonpenetration sham acupuncture group, the “Streitberger” needles (Asiamed Inc.) with blunt tips were placed at the two acupoints and were quickly removed afterwards, which only simulated the feeling of a needle (Xie et al., 2013). Procedures, electrode placements, and other treatment settings were the same in the electroacupuncture group but without any electrical output, or needle manipulation for “de qi”. During this study, we only had one senior acupuncturist for treatment operation, who had more than 20 years’ experience in acupuncture.

### 2.2 Outcome measurements

Participants were assessed with all outcome measurements at baseline, after 5 sessions of treatment, after 10 sessions of treatment, and at a 3‐month follow up. We used the Northwick Park Neck Pain Questionnaire (NPQ) as our primary assessment, and the 3‐month follow up as our primary endpoint. Due to the limited association between imaging diagnosis and neck pain symptoms, patient‐reported outcome measures (PROMs) have been proposed as a critical component for evaluating and monitoring neck pain. The NPQ scale as one of the PROMs has good validity, excellent internal consistency, and good reliability for neck pain measurement (Bobos et al., 2018; Sim et al., 2006). The NPQ scale with score 0–100 included nine items: the degree and duration of pain, symptoms including numbness, sleep and daily activities, and quality of life aspects. Higher scores represent worse neck pain. We define clinically significance as a 25% between‐group difference, based on the previous study by Sim et al. (2006).

We used the Short‐form McGill Pain Questionnaire (SF‐MPQ) and Pain threshold as the secondary outcomes. The SF‐MPQ includes pain rating index, present pain intensity, and visual analog scale (VAS, 0–10cm), which has good validity for chronic pain as well (Lovejoy et al., 2012). We also analyzed the VAS score separately from SF‐MPQ as another outcome measurement. To assess the pain threshold (PT), a semi‐objective outcome for neck pain, we placed the anode electrode of the device on the neck pain area, and the cathode on the leg (EP601C, East China Normal University of Science and Technology). With a gradually increasing current stimulation (0–2 mA), the patient pressed a stop button as soon as they felt pain. The average of three times of current strength (mA) was recorded as PT score (Defrin et al., 2005).

### 2.3 Blinding

All participants were asked if: they Strongly believe the treatment is real acupuncture (1), Somewhat believe the treatment is real acupuncture (2), Somewhat believe the treatment is placebo (3), Strongly believe the treatment is placebo (4), or Don't know (5). Instead of assessing blinding three times as described in our previous protocol paper, we only assessed blinding twice: after the last session of stimulation and at the 3‐month follow‐up visit. Because frequently asking participants to guess the intervention they received may increase the contextual effect and risk of unblinding (Hafliðadóttir et al., 2021). This protocol deviation was modified before the first participants enrolled. We used Bang's blinding index to assess the blinding, which scaled to an interval of −1 to 1 (1 being complete lack of blinding, 0 being consistent with perfect blinding, and −1 indicating opposite guessing) (Bang et al., 2004).

### 2.4 Statistical analysis

According to the NPQ results of a previous acupuncture study, which used NPQ scale as primary outcomes (Liang et al., 2011), we estimated a sample size of 29 subjects per group to provide 80% power to detect a between‐group difference of 4.14, assuming a standard deviation of 5.5 and a two‐sided significance level of 0.05. To compensate for a 10% loss of follow‐up rate, 33 subjects per group (total 169 subjects) were recruited.

The primary outcome was analyzed according to the modified intention‐to‐treat principle. The changes from baseline, 5 sessions after treatment, 10 sessions after treatment to 3‐month follow‐up were analyzed by fitting a mixed‐effect model with repeated‐measures approach. We used the baseline as a covariate; treatment, visit, and treatment × visit interaction as a fixed effect; and subject‐specific effects as random effect. Shapiro–Wilk normality test was used to test the distribution. For other continuous variables, comparisons between treatment groups were assessed using the *t* test or Wilcoxon rank‐sum test as appropriate. Categorical variables were compared using the Fisher's exact test or Wilcoxon rank‐sum test as appropriate. We used last observation carried forward to deal with missing data under the missing‐at‐random assumption. All statistical analyses were performed using Stata MP 14.0, Texas with two‐sided significance level of 0.05, which was then corrected for multiple testing using Bonferroni's correction with 5 comparisons, yielding a corrected alpha of 0.01.

## 3 RESULTS

**FIGURE 2:**
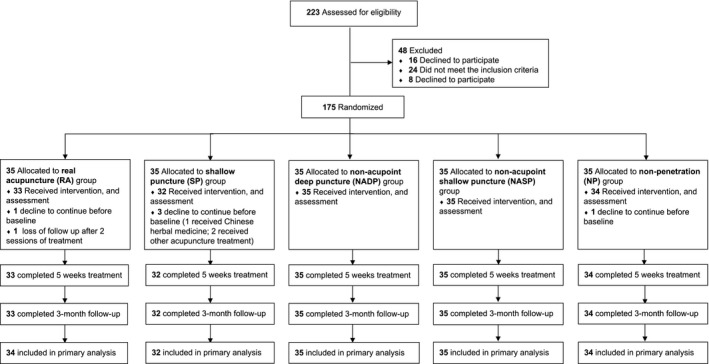
Consort flow diagram

We recruited a total of 203 neck pain patients from August 15th 2015 to August 15th 2018, and 175 of them were randomly assigned to the five groups, namely, electroacupuncture group (abbreviated EA, *n* = 33), shallow puncture group (SP, *n* = 32), nonacupoint deep puncture group (NADP, *n* = 35), nonacupoint shallow puncture group (NASP, *n* = 35), and nonpenetration group (NP, *n* = 34). Among the randomized patients, 169 completed the study (Figure 2). Last observation carried forward was used for the missing data in 1 participant. We excluded another five subjects who dropped out before baseline. There was no significant difference for age, gender, the time since the onset of neck pain, the affected side, previously received acupuncture, medication used, and baseline assessments between groups (Table 1). Six participants stopped the NASID during the study (2 in EA, 1 in NADP, 1 in NASP, 1 in NP). None of the participants reported taking new drugs or other interventions for pain treatment. There was no correlation between drug changes and the outcome results. No severe adverse events were reported during the study (Table S1).

**TABLE 1:** Demographic and baseline characteristics

	Electroacupuncture (34)		Shallow puncture (32)		Nonacupoint puncture (35)		Nonacupoint shallow puncture (35)		Nonpenetration acupuncture (34)	
Age (years), mean (SD)	41.15	(14.36)	33.87	(14.38)	44	(12.77)	39.77	(14.97)	40.03	(14.42)
Female sex, *N* (%)	29	(85%)	25	(78%)	32	(91%)	32	(91%)	27	(79%)
Time since neck pain (month), mean (SD)	65.85	(49.89)	52.25	(55.67)	71.14	(63.77)	59.34	(64.79)	46.56	(40.79)
Time of lower head per day (hours) includes, mean (SD)	5.62	(2.5)	6.27	(2.31)	6.49	(2.86)	6.46	(2.93)	5.99	(2.13)
Lasting hours per week (hours), mean (SD)	5.66	(7.82)	5.66	(10)	3.96	(5.93)	4.33	(6.3)	3.79	(6.5)
Frequency of pain per week (episodes), mean (SD)	3.56	(2.3)	3.53	(2.2)	3.63	(2.4)	2.91	(1.93)	2.97	(1.22)
Previously received acupuncture, *N* (%)	19	(55.88)	20	(62.5)	19	(54.29)	21	(60)	22	(64.71)
Medication used (NSAID), *N* (%)	3	(11.76)	3	(9.38)	4	(11.43)	2	(5.71)	3	(8.82)
NPQ score, mean (SD)	31.19	(13.37)	29.41	(12.76)	32.22	(11.12)	26.35	(14.88)	32.42	(13.76)
SF‐MPQ, mean (SD)	16.55	(6.84)	16.58	(7.23)	16.9	(7.66)	14.89	(7.38)	18.25	(7.56)
VAS, mean (SD)	5.63	(1.27)	4.53	(1.39)	5.59	(1.55)	5.16	(1.37)	4.61	(1.24)
PT (mA), mean (SD)	157.91	(44.9)	148.47	(41.69)	187.91	(70.33)	172.08	(58.92)	140.69	(49.43)

Note

Time of lower head per day (hour) include the time of working with computer, watching phone, writing, and reading; Frequency of pain per week is measured by the episodes per week.

Abbreviations: NPQ, Northwick Park Neck Pain Questionnaire; PT, pain threshold; SF‐MPQ, Short‐form McGill Pain Questionnaire;VAS, visual analog scale.

### 3.1 NPQ

**FIGURE 3:**
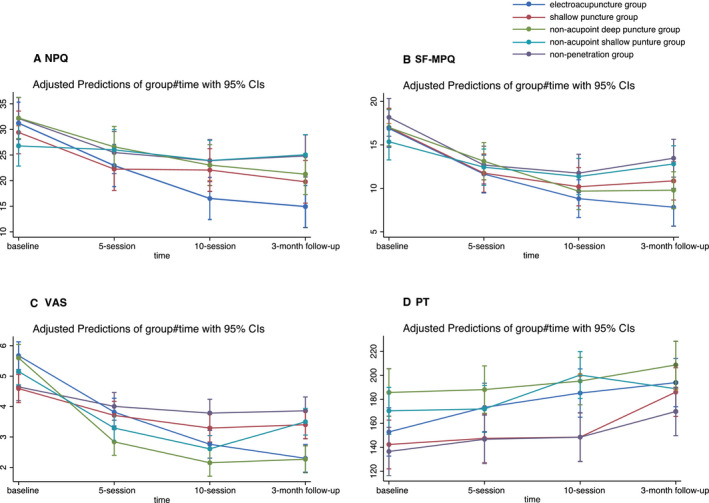
In this figure it is presented the observed scores of the outcome assessments during the study; error bars represent 95% CIs. (a) In the result of NPQ score, the electroacupuncture had the highest decrease. Despite there was no significant difference between electroacupuncture and non‐acupoint deep puncture, the electroacupuncture still significantly improved more than the other sham control groups. (b, d) In the SF‐MPQ and PT result, there were no significant difference between all groups. (c) The VAS had the same result as NPQ. NPQ, Northwick Park Neck Pain Questionnaire; PT, pain threshold; SF‐MPQ, Short‐form McGill Pain Questionnaire; VAS, visual analogue scale

We compared the effect of electroacupuncture with the NPQ scale across the five groups. The reduction of NPQ scores were different between groups over the study time (Chi^2^ = 69.83, df = 12, *p < 0.001). Except for the NASP group, all the other groups had a significant reduction in neck pain at the 3‐month follow up (Table 2, Figure 3a). All these reductions kept the linear trend (*p < 0.001). The EA group had higher improvements than the SP, NASP, and NP groups and a similar improvement with the NADP group (Table 2). Most interestingly, the EA, SP, and NADP groups achieved clinically significant reductions with more than 25% of reduction in NPQ after 10 sessions (52.2%, 32.78%, 34.04%; respectively).

**TABLE 2:** Primary and secondary outcomes

	Electroacupuncture (34)	Shallow puncture (32)	Nonacupoint puncture (35)	Nonacupoint shallow puncture (35)	Nonpenetration acupuncture (34)	*p* value between groups
NPQ						
Change at 1‐month follow‐up, adjusted mean (95%CI)	**−16.28** (−19.16 to −13.39)	**−9.64** (−12.57 to −6.71)	**−10.97** (−13.78 to −8.17)	−1.73 (−4.49 to 1.03)	**−7.33** (−10.21 to −4.44)	<0.001*
Change after 10 EA treatments, adjusted mean (95%CI)	**−14.70** (−17.59 to −11.82)	**−7.35** (−10.28 to −4.42)	**−9.19** (−11.99 to −6.39)	**−2.85** (−5.61 to −.09)	**−8.23** (−11.12 to −5.35)	<0.001*
Change after 5 EA treatments, adjusted mean (95%CI)	**−8.27** (−11.16 to −5.39)	**−7.16** (−10.09 to −4.24)	**−5.63** (−8.42 to −2.83)	−0.76 (−3.52 to 2.00)	**−6.66** (−9.54 to −3.77)	0.0082*
SF‐MPQ						
Change at 1‐month follow‐up, adjusted mean (95%CI)	**−9.06** (−11.86 to −6.27)	**−6.16** (−8.99 to −3.32)	**−7.21** (−9.93 to −4.5)	**−**2.56 (−5.23 to 0.12)	**−4.71** (−7.5 to −1.91)	0.1511
VAS						
Change at 1‐month follow‐up, adjusted mean (95%CI)	**−3.37** (−3.97 to −2.77)	**−1.19** (−1.80 to −.58)	**−3.33** (−3.91 to −2.74)	**−1.65** (−2.23 to −1.08)	**−0.78** (−1.39 to −.18)	<0.001*
PT						
Change at 1‐month follow‐up, adjusted mean (95%CI)	**41.12** (19.41 to 62.83)	**43.70** (21.66 to 65.74)	**22.96** (1.89 to 44.04)	18.45 (−2.34 to 39.23)	**33.35** (11.64 to 55.06)	0.2862

Note

The significance of bold values is *p*< 0.05.

Abbreviations: NPQ, Northwick Park Neck Pain Questionnaire; PT, pain threshold; SF‐MPQ, Short‐form McGill Pain Questionnaire; VAS, visual analog scale.

* Statistically significant difference (*p* < 0.01).

The EA group showed a higher reduction of NPQ than the NASP group as early as five‐sessions of stimulation (Table 3). Moreover, both NADP and SP groups had significantly higher improvements than the NASP group (*p* < 0.001, Table 4). Compared with the NP group, the electroacupuncture had 0.89 of effect size in Cohen's d test. The effect size of EA when comparing with the NASP group, which had the least observed effect, was 1.58 in Cohen's d test.

**TABLE 3:** Comparisons between electroacupuncture and sham control groups, respectively

	Electroacupuncture versus shallow puncture	Electroacupuncture versus nonacupoint deep acupuncture	Electroacupuncture versus nonacupoint shallow puncture	Electroacupuncture versus nonpenetration acupuncture
NPQ				
Change over study, Wald Chi^2^, *p*‐value	19.43, **0.0002** *	8.61, 0.0350	62.20, **<0.001** *	156.98, **<0.001** *
Change after 10 EA treatments, *F* value, *p*‐value	5.75, **0.0041** *	3.08, 0.0493	15.77, **0.0000** *	4.53, 0.0125
Change after 5 EA treatments, *F* value, *p*‐value	0.19, 0.6658	1.26, 0.2658	12.45, **0.0008** *	0.53, 0.4676
SF‐MPQ				
Change over study, Wald Chi^2^, *p*‐value	4.78, 0.1886	1.01, 0.7983	21.64, **<0.001** *	7.99, 0.0462
VAS				
Change over study, Wald Chi^2^, *p*‐value	37.69, **<0.001** *	7.91, 0.0479	27.21, **<0.001** *	51.24, **<0.001** *
PT				
Change over study, Wald Chi^2^, *p*‐value	5.98, 0.1126	2.96, 0.3982	3.71, 0.2945	2.24, 0.5242

Note

We used mix‐effect model to analysis the difference between electroacupuncture and sham acupunctures, respectively. The significance of bold values is *p* < 0.01.

Abbreviations: NPQ, Northwick Park Neck Pain Questionnaire; PT, pain threshold; SF‐MPQ, Short‐form McGill Pain Questionnaire; VAS, visual analog scale.

* Statistically significant difference (*p* < 0.01).

**TABLE 4:** Comparison between sham acupuncture groups

	Shallow puncture and nonacupoint deep puncture	Shallow puncture and nonacupoint shallow puncture	Shallow puncture and nonpenetration acupuncture	Nonacupoint deep puncture and nonacupoint shallow puncture	Nonacupoint deep puncture and nonpenetration acupuncture	Nonacupoint shallow puncture and nonpenetration acupuncture
NPQ						
Change over study, WaldChi^2^, *p*‐value	3.10, 0.3771	19.58, **0.0002** *	2.54, 0.4687	22.61, **<0.001** *	5.18, 0.1588	12.41, **0.0061** *
SF‐MPQ						
Change over study, WaldChi^2^, *p*‐value	2.07, 0.5579	6.40, 0.0938	1.09, 0.7797	7.61, 0.0548	4.05, 0.2559	2.47, 0.4811
VAS						
Change over study, WaldChi^2^, *p*‐value	44.70, **<0.001** *	12.43, **0.0061** *	1.55, 0.6717	16.06, **0.0011** *	48.62, **<0.001** *	17.13, **0.0007** *
PT						
Change over study, Wald Chi^2^, *p*‐value	3.88, 0.2746	12.46, **0.0060** *	46.51, **<0.001** *	3.51, 0.3190	0.64, 0.8868	5.45, 0.1414

Note

We used mix‐effect model to pairwise compare the sham acupunctures. The significance of bold values is *p* < 0.01.

Abbreviations: NPQ, Northwick Park Neck Pain Questionnaire; PT, pain threshold; SF‐MPQ, Short‐form McGill Pain Questionnaire; VAS, visual analog scale.

* Statistically significant difference (*p* < 0.01).

### 3.2 SF‐MPQ

Differing from the results of NPQ, there were no statistical differences in SF‐MPQ score changes between the sham groups over time (Chi^2^ = 16.96, *p* = 0.1511) (Table 2, Figure 3b). The EA group had the highest reduction in SF‐MPQ score at 3‐month follow up (adjusted mean = −9.06, *p < 0.001, [95%CI, −11.16 to −5.39]) (Table 2). However, only when compared with the NASP group, did the EA group show a significant difference (Chi^2^=21.64, *p < 0.001) (Table 3).

### 3.3 VAS

In addition, we analyzed the VAS score in SF‐MPQ separately. The result of VAS score showed a significant difference between groups (Chi^2^ = 89.78, *p < 0.001) (Table 2, Figure 3c). The EA group had a higher improvement than the other groups, except compared with the NADP group (with Bonferroni correction: Chi^2^ = 7.91, *p* = 0.0479) (Table 3). Moreover, the NADP group had significant higher reduction than NASP group. And both NADP and NASP groups were significantly better than SP and NP groups (Table 4). Interestingly, VAS scores in the NASP group significantly increased by 0.89 (*p = 0.002, [95% CI = 0.31 to 1.46]) during the follow up.

### 3.4 PT

The changes of PT among groups also did not show any significant difference (Chi^2^=14.23, *p* = 0.2862) over the study time. Similar to the NPQ and VAS results, all groups had significantly increased PT score, which related to the improvement of neck pain, except for NASP group (adjusted mean = 18.45, [95%CI, −2.34 to 39.23]) (Table 2, Figure 3d). However, the EA group in PT assessment did not show any superiority over the other groups (Table 3).

There were no significant correlations between PT and clinical scales (with NPQ: coef = −0.31, *p* = 0.366; SF‐MPQ: coef = −0.68, *p* = 0.22; VAS: coef = −1.09, *p* = 0.593).

### 3.5 Assessment of blinding

We used Bang's blinding index (BBI) to evaluate the blinding, as the patient's expectation is a key component of the placebo effect. Table S2 presents the percentage of participants in blinding scale. The results of BBI after the last session of stimulation were similar to the 1‐month follow up. In the EA condition, 79% (26 out of 33) participants strongly believed they received real acupuncture (BBI = 0.80, [95%CI, 0.68 to 0.92]). The comparisons of blinding rates showed no significant difference between sham control groups, as assessed by chi‐square test (*p* = 0.415). All sham control groups were successfully blinded (shallow puncture group: BBI = −0.015, [95%CI, −0.22 to 0.19]; nonacupoint shallow puncture group: BBI = 0.01, [95%CI, −0.17 to 0.19]; and nonpenetration group: BBI = 0.02, [95%CI, −0.15 to 0.21]), while 29% (10 out of 35) participants in the nonacupoint deep puncture (sham) group had higher probability of guessing that they received real acupuncture (BBI = −0.24, [95%CI, −0.42 to −0.07]).

## 4 DISCUSSION

Our study, for the first time, compared the specific and nonspecific effects among four different types of sham acupuncture stimulation with neck pain patients. Most notably, all of these groups, except nonacupoint shallow puncture, had significant improvements in all of our results (NPQ, SF‐MPQ, and PT). Despite the electroacupuncture (EA) group improving 52% in the NPQ scale, the differences in all outcome measurements with nonacupoint deep puncture was still not significant. Only when compared with nonacupoint shallow puncture, EA had significant improvement in clinical outcomes. Such dramatic variations in the analgesia effect produced by these sham acupunctures may lead to significant heterogeneity between the results of electroacupuncture efficacy studies using different controls.

Nevertheless, a recent multicenter study EA produced superior improvement than shallow puncture and nonpenetration group only in NPQ and VAS scale.

In our study, the magnitude of placebo response showed high variability between the four different types of sham controls, which has also been found in other pain studies (Hrobjartsson & Gøtzsche, 2001; Vase et al., 2002, 2009). A recent multicenter study of acupuncture treatment in neck pain found a wide variation of effect size among centers (Cohen's d 5 0.01–2.19) (Chen et al., 2021). In clinical practice, acupuncture analgesia may be explained by the combination of therapeutic effect and placebo response, which are also referred to as contextual effect (Hafliðadóttir et al., 2021). The placebo response is defined as all within‐group improvements that occur following administration of an inactive treatment and is attributable to both nonspecific effects such as disease spontaneous improvement, regression toward the mean and specific placebo mechanisms (placebo effect) (Evers et al., 2018). The mechanism of acupuncture placebo effect results in the release of neuropeptides, which reduce the activation in brain areas, such as the spinal cord, periaqueductal gray, rostroventral medulla, medial thalamus, and dorsal anterior cingulate cortex (Colloca, 2019). Vase et al. concluded that placebo analgesia reflects anti‐hyperalgesic mechanisms by exerting inhibitory effects on wind‐up‐pain intensity, central sensitization, secondary hyperalgesia, and ongoing clinical pain (Baron et al., 2013; Latremoliere & Woolf, 2009; Li et al., 1999; Vase et al., 2016). Interestingly, patients with higher response to placebo analgesia effect may have greater ability for pain modulation, which may result in a higher response to therapeutic effect (Lund et al., 2014). Thus, some studies used placebo effects to assess individual endogenous pain modulatory systems (Vase et al., 2016). In the last decade, the magnitude of the placebo effect seems to have increased (Häuser et al., 2011). Other studies showed that the average placebo analgesia effect in pain has Cohen's d above 0.8 (Petersen et al., 2014; Vase et al., 2002, 2009), thereby making it difficult to distinguish the placebo response from therapeutic effect, especially for acupuncture treatment.

The effects of penetrating versus nonpenetrating sham acupuncture have been discussed for a long time. In order to successfully blind patients, clinical trials need to have sham acupuncture controls. Penetrating sham controls induce the feelings of needles and can easily blind participants, thereby more acupuncture RCTs choose this type of sham control (Vickers et al., 2018). In Chen's multi center neck pain study and Pai's shoulder pain study, they both used shallow puncture as sham controls. Although both acupoint and nonacupoint shallow puncture controls in their studies had significant placebo response, the acupuncture/dry needle still showed a clinically significant superior efficacy in treating neck/shoulder pain, which were consistent with our results (Chen et al., 2021; Pai et al., 2021). Interestingly, in our study the nonacupoint shallow puncture had even less placebo response when compared with the nonpenetration acupuncture. However, meta‐analysis showed that among studies with successful blinding, none showed significant difference between acupuncture and penetrating sham control, while 28.6% achieve clinical significance by using nonpenetrating sham control (Chen et al., 2016; Vickers et al., 2018). Thus, lots of studies showed that superficial needling or deep needling at nonacupoints were as effective as real acupuncture in reducing pain outcomes, and all these methods can induce analgesia effect (Chen et al., 2019). Therefore, depending on the puncture depth and location (nonacupoint), the penetrating sham controls can have significantly different placebo response in treating pain.

The physiological mechanisms of penetration sham controls (including shallow puncture and nonacupoint deep puncture) may be the same as real acupuncture. Previous studies suggest that acupuncture analgesia may work through diffuse noxious inhibitory controls (DNIC), which is the response from a painful stimulus that inhibits pain from another stimulus (i.e., pain‐inhibiting‐pain effect) (Bing et al., 1990). However, another study of healthy individuals investigating the analgesic effect of acupuncture found that the effect can be explained by the improvement of tactile acuity via somatotopically specific structural primary somatosensory cortex neuroplasticity, which can also be induced by penetrating sham controls (Cao et al., 2020). Kagitani et al. concluded that the acupuncture analgesia effect was mediated by the activation of afferent nerve fibers innervating both the skin and muscles (Kagitani et al., 2010). This study suggested that regardless of whether shallow or deep penetration were applied, somatic afferent fibers, such as thick myelinated Aα and Aβ (group I and II), thin myelinated Aδ (group III), and thinner unmyelinated C (group IV) are stimulated by acupuncture and are involved in its effects. Stimulating either the abdominal skin or the underlying muscles produce a similar response (Sato et al., 1996), especially when the nonacupoint were only 1.5–2.5 cm apart to the true acupoints in nearly all of the acupuncture studies. Moreover, we found in our blinding results that participants in the nonacupoint deep puncture (NADP) group were more likely to believe they received real acupuncture. This may suggest that NAPD also produces similar psychological analgesic effect as EA.

Nonpenetrating sham acupuncture has a different pathway of neuromodulation than penetrating sham acupuncture. Because there is no stimulation in nonpenetration sham acupuncture, many studies conclude that it produces only pure placebo response or contextual effect. However, our results showed that it led to similar effects in SF‐MPQ and PT as electroacupuncture (Tables 3 and 4). Our findings are supported by Harris et al. studies, which suggested that while active acupuncture evoke both short‐term and long‐term μ‐opioid receptors binding potential (MOR BP), and only a small reduction of MOR BP was observed in nonpenetrating sham group, the changes in SF‐MPQ score were still similar between the active group and nonpenetrating sham groups (Harris et al., 2009). Nonpenetrating sham acupuncture induces the contextual and treatment cues which trigger a set of psychoneurobiological changes as placebo response (Price et al., 2008). The expected pain levels and emotional feelings of relief contribute to the placebo response in pain, which enhance the descending pain inhibitory pathways, including core rACC‐amygdala‐PAG‐ rostroventral medulla–spinal cord connection (De Pascalis et al., 2002; Zubieta et al., 2005, 2006). A previous study showed placebo analgesia reduced the neural activity in the spinal cord (Eippert et al., 2009). Wager et al. found that placebo treatment produces analgesia by altering the expectation of participants, which is related to decreased brain activity in pain‐sensitive brain regions, including the thalamus, insula, and anterior cingulate cortex, and was associated with increased activity during anticipation of pain in the prefrontal cortex (Wager et al., 2004). Thus, the analgesia effect in nonpenetrating sham stimulation was mainly induced by a psychoneurobiological mechanism, which differs from penetrating sham controls.

Selection of the outcome assessment of pain is another crucial factor for acupuncture clinical research. The results of the four outcome scales had a lot of heterogeneity among groups. In our study, the NPQ and VAS scales are more sensitive in identifying the electroacupuncture effect than SF‐MPQ and PT. This is also consistent with Harris’ study, which used SF‐MPQ as the clinical outcome, and observed no significant difference between the acupuncture group and nonacupoint nonpenetration sham group, although real acupuncture had significantly activated the μ‐opioid receptors compared with the sham (Harris et al., 2009). Although NPQ and SF‐MPQ are both holistic measurements for neck pain, the acupuncture effects on each of them differed significantly. Previous reviews found that the placebo effect was greater in studies using continuous subjective outcomes and for the treatment of pain (Hrobjartsson & Gøtzsche, 2001). The common conclusion in clinical research is that, if the treatment does not produce superior efficacy to sham, it is ineffective and only operating via placebo effect (Chen et al., 2019).

However, our study suggests this conclusion may be superficial, as the observed efficacy of acupuncture may differ according to the selected outcome measurement.

The placebo response can be magnified by increasing the dosage of sham stimulation. Table 2 and Figure 3 showed that with the number of treatments increase, the overall effect of sham control increases as well, which may be driven by the increase in placebo‐mediated analgesia. Colloca's review demonstrated that the dose‐extending placebos that harness the body's capacity to create learned conditioned responses and pharmacological memories can in turn trigger the activation of opioid and nonopioid endogenous pain modulatory systems (Colloca, 2019). Moreover, unlike other penetration sham controls, the analgesia effect in nonacupoint shallow puncture and nonpenetration group decreased during the follow up. This may indicate that sham puncture with a deeper puncture depth or puncture at the acupoints (regardless of depth) could induce the specific therapeutic effect or nonspecific physiological analgesia effect of acupuncture.

A limitation of our study is that 79% of the patients who received the electroacupuncture correctly guessed the intervention they received, which may be due to the manipulation of acupuncture (*de qi* sensation and feeling of electrical current) or previous exposure to acupuncture (we did not exclude participants who received acupuncture 3 months before the enrollment). This may inflate the placebo response of electroacupuncture. Moreover, 29% of participants in nonacupoint deep puncture strongly believed they received active stimulation which may also have significantly biased the result. However, blinding for the active stimulation is a common limitation in acupuncture studies, because of the strong sensation delivered by the needles and current (Hinman et al., 2014). Other electrical stimulation studies face a similar challenge, for instance, in transcranial direct current stimulation, transcranial magnetic stimulation, and transcutaneous electric nerve stimulation, it is difficult to blind participants in active stimulation groups (Greinacher et al., 2019; O’Connell et al., 2012; Turi et al., 2019; Wallace et al., 2016). Turner reported that 78.1% of participants guessed correctly when they received 1mA tDCS, which was similar to the percentage of failure in the active group blinding in our study (Turner et al., 2021). Therefore, blinding for the active group in research about many types of electrical‐current stimulation interventions is generally a challenge.

Furthermore, the method and time points of blinding assessment also limited our study. Although many participants in active groups successfully identified the stimulation they received after the last sessions of stimulation, and at the 1‐month follow up, our assessor reported that some of these participants suspected they received the sham during the study. Turner's study also concluded that the “end‐of‐study guess” does not reflect the participants’ sensitivity to the presence of stimulation, which may be due to the confusion in distinguishing between “sham” and “active,” poor memory of the prior sessions, influence by prior experience or knowledge of treatment, or subtle priming by the experimenters (Rabipour, 2018, 2019). Therefore, further research of blinding evaluation methods is needed, especially for stimulation research.

Another limitation is that we only recorded 1‐week baseline assessment, considering patient adherence. Although most of the pain studies record the pain diary around for 1‐week, longer baseline assessment could provide a more stable baseline score (Hinman et al., 2014).

In conclusion, electroacupuncture has clinically significant efficacy in the treatment of neck pain, despite the nonacupoint deep puncture producing similar analgesia effects. The most important implication of our results is that the variability of placebo response among different types of sham controls, which depends on the puncture depth and location (nonacupoint), limits the assessment of effect size of real acupuncture. Our study may shed a new light on the predominant underlying mechanisms among different types of sham acupuncture controls, which can help with interpretations of the effect of acupuncture in other studies.

## AUTHORS’ CONTRIBUTION

Drs Dian Zeng, Jianhua Liu, and Wenbin Fu had had full access to all of the data in the study and take responsibility for the integrity of the data and the accuracy of the data analysis. D. Zeng, J. Liu, and W. Fu. contributed to concept and design. D. Zeng, J. Liu, N. Xu, X. Yan, H. Deng, J. Li, J. Xiao, J. Yuan, and J. Huang contributed to acquisition, analysis, or interpretation of data. D. Zeng, and J. Liu contributed to drafting of the manuscript. All authors contributed to critical revision of the manuscript for important intellectual content. D. Zeng contributed to statistical analysis. J. Liu, W. Fu, and N. Xu obtained funding.

## Supporting information

Table S1–S2
