# Supplementary material for: Placebo response varies between different types of sham acupuncture: A randomized double‐blind trial in neck pain patients
Source: Eur J Pain. 2022 Feb 19;26(5):1006–20. doi: 10.1002/ejp.1924 (PMC9305463; doi:10.1002/ejp.1924)
Supplement: Supplementary file 1 — Table S1–S2 [file EJP-26-1006-s001.docx]

**Supplementary table 1** Treatment related adverse events

|  | Electroacupuncture | Shallow puncture | Non-acupoint deep puncture | Non-acupoint shallow puncture | Non-penetration acupuncture |
| --- | --- | --- | --- | --- | --- |
| Severe adverse event | 0 | 0 | 0 | 0 | 0 |
| Sharp pain | 2 | 0 | 1 | 1 | 0 |
| Sensitive skin | 0 | 1 | 0 | 0 | 1 |
| Tingling | 1 | 0 | 1 | 0 | 0 |
| Tiredness | 0 | 1 | 0 | 0 | 1 |
| Pain in other areas | 0 | 0 | 0 | 1 | 1 |

Number of participants reported adverse event in each group.

**Supplementary table 2** result of Bang’s blinding index

|  | Strong acupuncture | Somewhat acupuncture | Somewhat sham acupuncture | Strong sham acupuncture | Don’t know |
| --- | --- | --- | --- | --- | --- |
| After last session of treatment | | | | | |
| Electroacupuncture, N (%), (total=33) | **24 (73%)** | **3 (9%)** | 1 (3%) | 0 | **5 (15%)** |
| Shallow puncture, N (%), (total=32) | 7 (22%) | 6 (19%) | **6 (19%)** | 6 (19%) | **7 (22%)** |
| Non-acupoint deep puncture, N (%), (total=35) | 10 (29%) | **9 (26%)** | 7 (20%) | 2 (6%) | **7 (20%)** |
| Non-acupoint shallow puncture, N (%), (total=35) | 6 (17%) | 6 (17%) | 9 (26%) | 5 (14%) | 9 (26%) |
| Non-penetration acupuncture, N (%), (total=34) | 5 (15%) | 6 (18%) | 6 (18%) | 6 (18%) | 11 (32%) |
| At 1-month follow-up | | | | | |
| Electroacupuncture, N (%), (total=33) | **26 (79%)** | **2 (6%)** | 1 (3%) | 0 | **4 (12%)** |
| Shallow puncture, N (%), (total=32) | 7 (22%) | 6 (19%) | **7 (22%)** | 6 (19%) | **6 (19%)** |
| Non-acupoint deep puncture, N (%), (total=35) | 10 (29%) | **8 (23%)** | 7 (20%) | 2 (6%) | **8 (23%)** |
| Non-acupoint shallow puncture, N (%), (total=35) | 6 (17%) | 6 (17%) | 9 (26%) | 5 (14%) | 9 (26%) |
| Non-penetration acupuncture, N (%), (total=34) | 5 (15%) | 6 (18%) | 6 (18%) | 6 (18%) | 11 (32%) |

Bold fonts = the number of participants changed after 1-month follow-up
